# Comparison of Total Mesopancreatic Excision and Conventional Pancreaticoduodenectomy in the Surgical Treatment of Pancreatic Head Adenocarcinoma: Early Postoperative Outcomes

**DOI:** 10.3390/medicina61101725

**Published:** 2025-09-23

**Authors:** Tufan Egeli, Tarkan Unek, Mucahit Ozbilgin, Cihan Agalar, Anıl Aysal Agalar, Ilkay Tugba Unek, Caner Bektas, Gokce Kıran Kazancı, Berkay Sakaoglu, Emre Karadeniz, Ozgul Sagol

**Affiliations:** 1 Department of General Surgery, Dokuz Eylul University, Izmir 35330, Türkiye; tarkan.unek@gmail.com (T.U.); mucahitozbilgin@gmail.com (M.O.); cihanagalar@hotmail.com (C.A.); canrbkts@gmail.com (C.B.); drgokcekiran28@gmail.com (G.K.K.); 2 Department of Pathology, Dokuz Eylul University, Izmir 35330, Türkiye; anilaysalagalar@gmail.com (A.A.A.); ozgul.sagol@deu.edu.tr (O.S.); 3 Division of Medical Oncology, Department of Internal Medicine, Dokuz Eylul University, Izmir 35330, Türkiye; ilkaytagbaunek@gmail.com; 4 Department of General Surgery, Nusaybin State Hospital, Mardin 47300, Türkiye; sakaoglub@gmail.com; 5 Department of General Surgery, Bagcilar Training and Research Hospital, Istanbul 34200, Türkiye; karadeniz_emre@hotmail.com

**Keywords:** pancreatic ductal adenocarcinoma, pancreaticoduodenectomy, total mesopancreatic excision, postoperative complications, treatment outcomes

## Abstract

*Background and Objectives*: This study aimed to evaluate and compare the early postoperative outcomes of patients who underwent pancreaticoduodenectomy (PD) with total mesopancreatic excision (TMpE) versus conventional pancreaticoduodenectomy (Co-PD) for pancreatic head ductal adenocarcinoma (PDAC). *Materials and Methods*: Patients who underwent PD for pancreatic head cancer between January 2021 and December 2024 in our clinic and had a pathological diagnosis of PDAC were included. Patients were stratified into two groups according to the surgical technique performed (TMpE-PD vs. Co-PD). Demographic characteristics and early postoperative clinicopathological data were compared between the groups. *Results*: A total of 41 patients were included: 17 (41.5%) underwent TMpE-PD and 24 (58.5%) underwent Co-PD. Demographic and clinicopathological parameters were comparable between the groups. Although not statistically significant, the TMpE-PD group demonstrated higher R0 resection rates (58.8% vs. 45.8%; *p* = 0.412) and greater lymph node yield (33.9 vs. 29.1; *p* = 0.757) compared to the Co-PD group. Overall postoperative complications were more frequent in the TMpE-PD group (82.4% vs. 63.4%; *p* = 0.034). A smaller pancreatic duct diameter was associated with an increased risk of postoperative complications in both groups, approaching statistical significance (*p* = 0.053). Multivariable logistic regression analysis revealed that the surgical technique was not an independent risk factor for postoperative complications (OR: 0.64; 95% CI: 0.14–2.83; *p* = 0.56). No direct correlation was found between resection margin status (R0 vs. R1) and the development of postoperative complications. *Conclusions*: TMpE demonstrated non-significant trends toward higher R0 resection rates and greater lymph node yield compared with conventional PD. These findings suggest possible oncological benefits without significantly increasing perioperative morbidity.

## 1. Introduction

Pancreaticoduodenectomy (PD, Whipple procedure) with curative intent (margin-negative/R0 resection), combined with adjuvant or neoadjuvant chemotherapy, currently represents the standard treatment for pancreatic ductal adenocarcinoma (PDAC) located in the pancreatic head [1,2,3,4,5,6]. However, only 15–25% of patients are eligible for surgical resection at the time of diagnosis [7]. Despite advances in surgical techniques over the years, R0 resection following PD is reported in only about 50% of cases [8]. Furthermore, local recurrence develops in approximately 80% of patients after PD, even in those considered to have undergone R0 resection, suggesting that true complete resection may not have been achieved [9,10]. Esposito et al. reported that the frequency of R1 resection margins in PD specimens increased from 14% to 76% when reassessed using standardized contemporary pathological evaluation methods [11]. Even in patients undergoing combined PD and chemotherapy for pancreatic head cancer, 5-year survival rates remain around 20–25% [12,13,14]. This poor outcome is attributed to early locoregional recurrence secondary to incomplete (non-curative) resections. Ghaneh et al. demonstrated that non-curative resections significantly reduce both overall and disease-free survival in patients with pancreatic cancer [15].

In 2007, Gockel et al. first introduced the concept of the “mesopancreas” [16]. The mesopancreas is defined as a structure located posterior to the pancreatic head, consisting of neural and lymphatic tissues extending along the mesenteric vascular axis. This region represents a major pathway for tumor spread in PDAC. Gockel emphasized that incomplete resections following PD often result from residual tumor deposits in this area and highlighted the necessity of performing a “total mesopancreatic excision (TMpE)” to achieve true curative resection. Supporting this view, Gadcke et al. demonstrated that the most frequent site of R1 resection after PD was the mesopancreatic region [17]. Subsequent studies have reported that PD with TMpE increases the likelihood of achieving true R0 resection, improves lymph node yield, and thereby confers a survival advantage [18,19,20].

Nevertheless, several controversies remain regarding TMpE. The anatomical definition of the mesopancreas has not reached universal consensus, with varying descriptions proposed by different authors [21]. Consequently, the optimal extent of TMpE remains a subject of debate. Moreover, different surgical approaches to TMpE have been suggested, and an internationally standardized technique has yet to be established [22]. Furthermore, some studies have reported increased morbidity, including refractory diarrhea and lymphorrhea following TMpE, which may delay the initiation of adjuvant chemotherapy [21,23].

The present study aimed to compare the early postoperative outcomes of pancreatic head PDAC patients who underwent PD with TMpE versus conventional PD (Co-PD) in our institution.

## 2. Patients and Methods

### 2.1. Ethical Approval and Patient Selection

The study was conducted in accordance with the Declaration of Helsinki and approved by the Institutional Review Board (or Ethics Committee) of Dokuz Eylul University (Protocol code: 2025/06-26 and date of approval: 19 February 2025). This study was approved by the institutional ethics committee. Patients who underwent pancreaticoduodenectomy (PD) for pancreatic head cancer in our clinic between January 2021 and December 2024 were retrospectively reviewed. Among these, only patients with a pathological diagnosis of pancreatic ductal adenocarcinoma (PDAC) were included. Informed consent was obtained from all patients involved in the study. Patients were stratified into two groups: those who underwent PD with total mesopancreatic excision (TMpE-PD) and those who underwent conventional PD (Co-PD). All procedures were performed by experienced surgeons within our hepatopancreatobiliary (HPB) surgery unit. While some surgeons preferred the Co-PD technique, others routinely performed TMpE-PD in all eligible cases.

### 2.2. Surgical Technique

**Figure 1:**
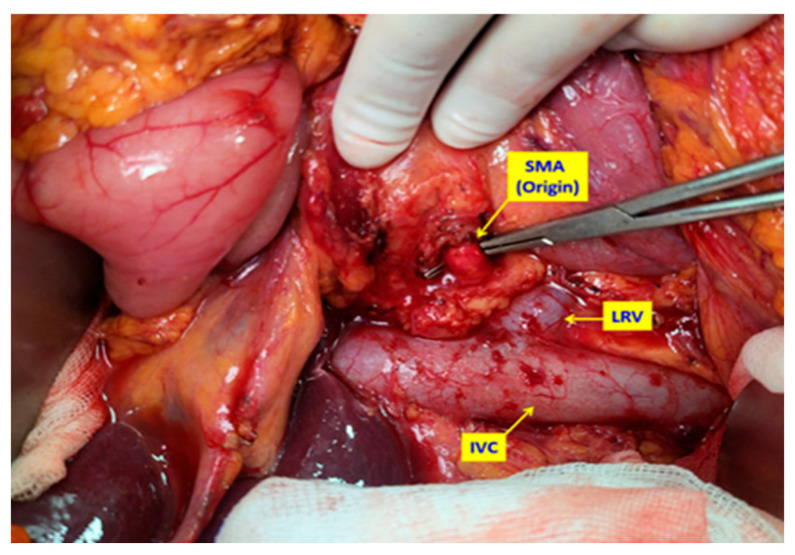
Intraoperative view of the first step: after the extended Kocher maneuver, the superior mesenteric artery (SMA) is dissected and encircled using the posterior artery-first approach, providing both proximal and distal vascular control and defining the dissection plane close to the arterial adventitia. LRV: Left renal vein IVC: Inferior vena cava.

**Figure 2:**
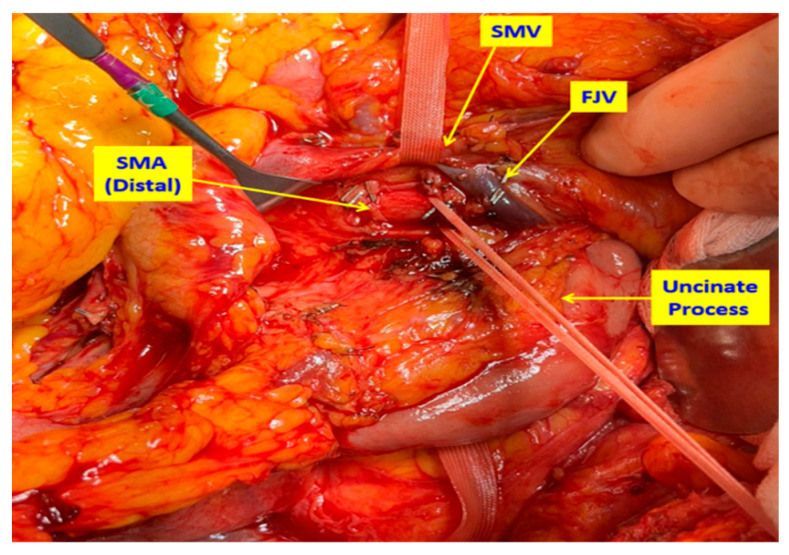
Intraoperative view of the second step: using the uncinate process approach, the distal portion of the superior mesenteric artery (SMA) is dissected and suspended. This maneuver ensures both proximal and distal vascular control of the SMA and facilitates safe en bloc mesopancreatic excision. FJV: First jejunal vein. SMV: Superior mesenteric vein.

In patients undergoing conventional PD (Whipple procedure), the standard, previously described surgical technique was applied [24]. In TMpE-PD cases, surgery was initiated with an artery-first approach. Following a wide Kocher maneuver, the superior mesenteric artery (SMA) was dissected posteriorly, encircled, and suspended. The dissection was extended distally via the uncinate process, allowing both distal and proximal control of the SMA (Figure 1 and Figure 2). Dissection proceeded in a caudo-cranial manner along the pancreatic border toward the SMA origin, at which point the artery was assessed for tumor invasion.

**Figure 3:**
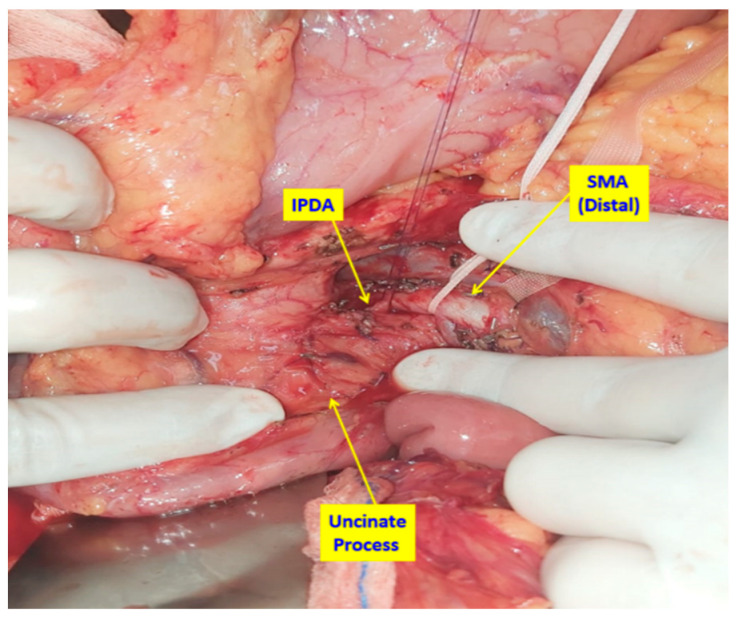
Intraoperative view of the dissection plane between the superior mesenteric artery (SMA) and the pancreas: the tissue is dissected as close as possible to the arterial wall, leaving the mesopancreatic tissue en bloc. The inferior pancreaticoduodenal artery (IPDA) is clearly visualized and prepared for ligation and division, highlighting the vascular control achieved during mesopancreatic excision.

If no invasion was detected, the tissue plane between the SMA and pancreas was dissected as close as possible to the arterial wall, leaving the mesopancreatic specimen en bloc. At this stage, the inferior pancreaticoduodenal artery (IPDA) and its branches were ligated and divided (Figure 3). The first jejunal vein (FJV) was preserved unless invaded by tumor, in which case it was ligated and divided. Similarly, branches of the first jejunal artery (FJA) were preserved unless infiltration was present, requiring ligation.

**Figure 4:**
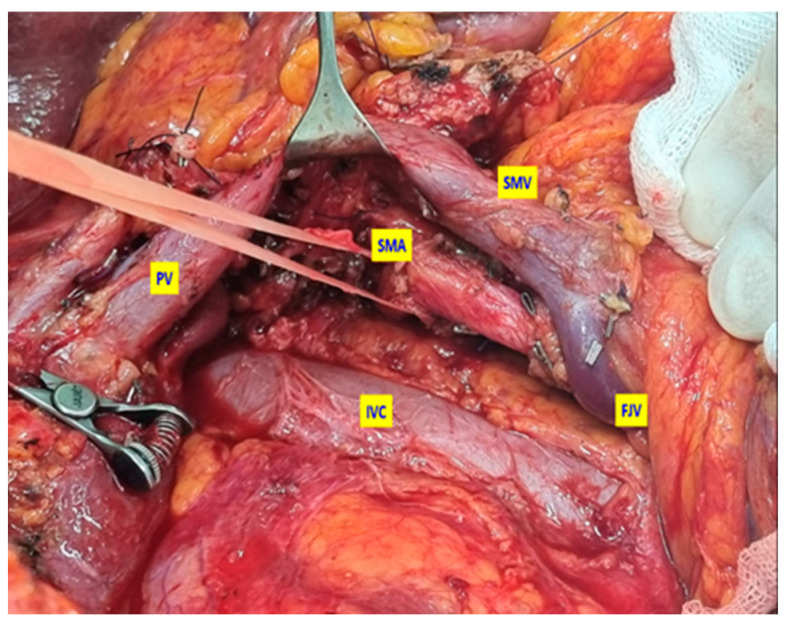
Intraoperative view of the surgical field after completion of the total mesopancreatic excision (TMpE), demonstrating complete clearance of the mesopancreatic tissue and the exposed adventitial surface of the superior mesenteric artery (SMA), confirming en bloc resection of the targeted anatomical unit. Intraoperative view of the surgical field after completion of the total mesopancreatic excision. SMV: Superior mesenteric vein; PV: Portal Vein; IVC: Inferior vena cava; FJV: First jejunal vein.

During the resection phase, the uncinate process was excised from the retroperitoneum with the mesopancreas included in the specimen (Figure 4).

In cases with SMV invasion, distal and proximal vascular control was established. Following temporary clamping, en bloc resection was performed, and vascular continuity was restored with reconstruction before proceeding with the remaining anastomoses.

In both groups, lymphadenectomy was performed according to the recommendations of the International Study Group of Pancreatic Surgery (ISGPS) [25]. Pancreaticojejunostomy was performed as an end-to-side anastomosis, with the pancreatic capsule sutured to the jejunal seromuscular layer using interrupted 3/0 silk sutures, and duct-to-mucosa anastomosis performed with interrupted 5/0 polydioxanone (PDS) sutures.

Pathological specimens were evaluated macroscopically and microscopically by pathologists specialized in HPB pathology. For macroscopic evaluation, pancreatic surgical margin, retroperitoneal surgical margin, common bile duct surgical margin, and vascular bed surgical margin sampling were performed on all specimens. Microscopic evaluation included tumor type and differentiation, tumor pathologic T and N stages (according to the AJCC 8th Edition), surgical margin status, the number of dissected lymph nodes, and the number of metastatic lymph nodes. All surgical margins were inked, axial slicing of the pancreatic head was performed, and margin reporting followed international consensus definitions. Although no central review was conducted, the pathologists assessing the specimens were blinded to the surgical technique (TMpE vs. Co-PD); therefore, margin evaluation was blinded with respect to surgical approach.

Demographic features, comorbidities, preoperative factors, tumor size, TNM stage, operative time, estimated blood loss, resection margin status (R0 vs. R1), lymph node yield, length of hospital stay, and postoperative complications were recorded. Postoperative complications were classified according to the Clavien–Dindo system [26], with grade III or higher defined as major complications. Postoperative pancreatic fistula (POPF) was classified according to ISGPS criteria [27].

This study evaluated the relationship between surgical techniques and resection margin status (R0/R1) with postoperative complications and investigated risk factors affecting the development of postoperative complications.

### 2.3. Statistical Analysis

Logistic regression models were built using purposeful selection methods by Hosmer and Lemeshow, with clinically relevant variables retained regardless of univariable significance. Confidence intervals (95% CI) were reported for all effect estimates.

All statistical analyses were performed using jamovi (The jamovi project, Sydney, Australia) software (version 2.6), an open-source statistical package [28]. Normality of distribution for continuous variables was assessed using the Shapiro–Wilk test and visual inspection of Q–Q plots. Continuous variables were expressed as mean ± standard deviation or median (interquartile range), and categorical variables as numbers (percentages). Group comparisons were performed using Student’s *t*-test or the Mann–Whitney U test for continuous variables, and Chi-square or Fisher’s exact test for categorical variables.

To evaluate prognostic factors associated with complications, clinicopathological variables were first analyzed by univariable methods. Variables with *p* < 0.25 and those of established clinical relevance were included in the multivariable logistic regression analysis. The purposeful selection method by Hosmer and Lemeshow was applied [29]. Coefficient changes and potential interactions were examined, and clinically significant variables were retained in the final multivariable model. Results were expressed as odds ratios (ORs) with 95% confidence intervals (CIs). Two-tailed *p* values < 0.05 were considered statistically significant.

## 3. Results

A total of 41 patients were included in the study, of whom 17 (41.5%) underwent TMpE-PD and 24 (58.5%) underwent Co-PD. Demographic and clinicopathological parameters were comparable between the two groups, with no statistically significant differences (Table 1). The median operative time was slightly longer in the TMpE-PD group compared to the Co-PD group (485 min vs. 423 min; *p* = 0.067). Venous resections and reconstruction were performed in five patients (2 TMpE-PD, 3 Co-PD). Only one of these patients (Co-PD) had received neoadjuvant therapy; in the remaining four cases, vascular resection had not been anticipated preoperatively.

Although not statistically significant, the TMpE-PD group showed higher R0 resection rates (58.8% vs. 45.8%; *p* = 0.412) and greater lymph node yield (33.9 vs. 29.1; *p* = 0.757) compared to the Co-PD group. Pancreatic duct diameter and gland texture were similar between groups.

Postoperative outcomes are summarized in Table 2. Overall complication rates were higher in the TMpE-PD group compared with the Co-PD group (82.4% vs. 63.4%; *p* = 0.034). However, when only surgical complications were considered, there was no significant difference between the two groups (70.6% vs. 58.5%; *p* = 0.187). Although not statistically significant, the incidence of major complications (Clavien–Dindo grade ≥ III) was lower in the TMpE-PD group compared to the Co-PD group (11.8% vs. 25.0%; *p* = 0.414).

Readmission within the first 30 days occurred in one patient (5.9%) in the TMpE-PD group and three patients (12.5%) in the Co-PD group (*p* = 0.628). Clinically relevant POPF (Grade B–C) rates were low and comparable between the groups (TMpE-PD 5.9% vs. Co-PD 8.3%; *p* = 1.000). Chylous fistula developed in two patients (14%) in the TMpE-PD group, whereas none occurred in the Co-PD group. Neither group experienced delayed gastric emptying, refractory diarrhea, or post-pancreatectomy hemorrhage affecting clinical course.

Overall, hospital mortality due to POPF occurred in two patients (4.8%), one from each group. Additionally, two further deaths (4.8%) occurred in the Co-PD group within 90 days postoperatively, both related to comorbidities.

Analysis of parameters potentially associated with postoperative complications revealed no statistically significant predictors (Table 3).

Only a smaller pancreatic duct diameter was identified as a risk factor approaching statistical significance (*p* = 0.053).

After the initial evaluation, variables with *p* values < 0.25, including sex (OR:2.57 CI 0.69–9.55, *p* = 0.158), pathological stage (OR:2.73 CI 0.72–10.27, *p* = 0.138), and pancreatic duct diameter (OR:0.75 CI 0.56–1.00, *p* = 0.053) were selected for further models. The adjusted model also included pancreatic gland texture (OR:3.00 CI 0.44–20.44, *p* = 0.262) due to its known clinical importance.

In multivariable logistic regression analysis, the surgical technique (TMpE-PD vs. Co-PD) was not found to be an independent risk factor for the development of postoperative complications (OR: 0.64; 95% CI: 0.14–2.83; *p* = 0.56). Similarly, no direct association was observed between resection margin status (R0 vs. R1) and complication development (Table 4).

### Discussion

Pancreatic ductal adenocarcinoma (PDAC) is one of the most aggressive malignancies, with approximately 80% of cases located in the pancreatic head [30,31]. In eligible patients, surgical resection with R0 margins remains the most effective treatment option [2,3,4,5]. Pancreaticoduodenectomy (PD) is the standard surgical procedure for pancreatic head adenocarcinomas, and technical advancements and modifications have been introduced over the years [32]. Nevertheless, even in patients undergoing curative-intent PD, early recurrence rates of 59.7–91.1% have been reported, most frequently attributed to R1 resection margins [33,34].

Total mesopancreatic excision (TMpE) has been proposed as a surgical technique with the potential to improve R0 resection rates and thereby reduce locoregional recurrence [16]. Excision of the mesopancreas during PD has been advocated to achieve more favorable oncological outcomes [17,35]. However, the definition of the mesopancreas remains controversial, with varying descriptions proposed by different authors [36,37,38]. As a result, no consensus has yet been reached regarding the optimal extent of TMpE. Furthermore, the absence of adequately powered randomized controlled trials limits the evidence on the efficacy and safety of TMpE [22].

Early reports of TMpE emphasized artery-first approaches to PD, particularly dissection beginning with the superior mesenteric artery (SMA) [18,20]. The artery-first approach offers several advantages, including early assessment of arterial invasion and avoidance of unnecessary resection in non-resectable cases [39]. For TMpE-PD, this strategy is regarded as technically advantageous. Studies comparing TMpE-PD with Co-PD have reported higher rates of R0 resection and greater lymph node yield with TMpE-PD. In a recent meta-analysis, Silva et al. also demonstrated that TMpE-PD achieved significantly higher R0 resection rates and lymph node retrieval compared with Co-PD [22].

Despite its potential oncological benefits, TMpE is associated with certain disadvantages. The procedure requires meticulous dissection along the adventitial plane of the SMA, which increases technical complexity and may prolong operative time. Furthermore, the more extensive dissection of the mesopancreatic tissue has the potential to increase perioperative morbidity. Extensive dissection around the SMA has been associated with potential morbidity, including refractory diarrhea and high-output chylous leakage due to neural and lymphatic injury [20,21,22,23]. For this reason, several authors have emphasized the need to avoid overly aggressive dissection. Refractory diarrhea not only increases morbidity but also delays the initiation of adjuvant chemotherapy [40,41,42]. Some reports have suggested that early use of antidiarrheal agents may help manage this complication [22,43]. In our series, no patients in the TMpE-PD group developed refractory diarrhea. In patients who developed diarrhea, dietary modifications, probiotics, and antidiarrheal medications were effective for symptom control. Chylous fistula occurred in two patients (14.8%) in the TMpE-PD group but in none of the Co-PD group, consistent with previous reports linking this complication to more extensive lymphatic dissection.

These findings highlight a central dilemma for surgeons: whether to accept an increased risk of postoperative morbidity—particularly refractory diarrhea—in exchange for potentially superior oncological clearance. The decision ultimately depends on defining the optimal extent of mesopancreatic dissection required for TMpE. Although multiple techniques have been described, no universally standardized and internationally accepted definition of the procedure exists to date [17,18,19,20,21,22,44,45,46].

Inoue et al. were the first to describe a systematic supracolic artery-first mesopancreatic excision for periampullary tumors, defining three levels of dissection of increasing extent [47]. They reported that TMpE-PD, compared with Co-PD, was associated with reduced operative time and blood loss. More recently, Nagakawa et al. proposed a refined TMpE technique based on four intense neurofibrous tissue (NFT) zones around the SMA (A, B, C, D areas), which provided a structured framework for dissection [48]. Compared with conventional PD, this approach achieved shorter operative times, reduced blood loss, and improved rates of R0 resection and lymphadenectomy.

In our institution, TMpE-PD is performed using a posterior and uncinate process combined artery-first approach, as described in the Methods section. The principle is to achieve early SMA control, assess resectability, and perform PD with en bloc removal of the mesopancreas. The extent of dissection is individualized intraoperatively, with the extension of resection margins determined when tumor infiltration is observed. Thus, oncological advantage is prioritized while balancing the relative risk of increased postoperative morbidity. We believe that in TMpE-PD, the extent of resection should be tailored to each patient based on intraoperative findings.

Considering that all tumors in this study were PDAC, our approach most closely resembles the level 2 dissection described by Inoue et al. Although statistical significance was not reached, TMpE-PD cases demonstrated trends consistent with the literature, including higher R0 resection rates and greater lymph node yields compared with Co-PD cases. In univariable analysis, overall complications were more frequent in the TMpE-PD group; however, when limited to surgical complications, no significant differences were observed. Moreover, major complications (Clavien–Dindo grade ≥ III) were more frequent in the Co-PD group. Multivariable logistic regression analysis demonstrated no significant differences in postoperative complication rates between the two groups. Similarly, no relationship was found between resection margin status (R0 vs. R1) and complication development. These findings suggest that more aggressive dissection to achieve R0 resection does not necessarily translate into higher postoperative morbidity. Among all cases, only a smaller pancreatic duct diameter approached significance as a risk factor for complications.

This study has several limitations. Its retrospective, single-center design and relatively small sample size limit the generalizability and statistical power of the findings. The retrospective, single-center design and relatively small sample size inherently limit causal inference and statistical power. The absence of propensity score matching may introduce selection bias. Different surgeons performed each technique, creating potential performance bias. Neoadjuvant therapy was underrepresented, reducing generalizability. Long-term outcomes (recurrence, survival) were not available due to short-term analysis. The regression model was exploratory and not validated.

## 4. Conclusions

Total mesopancreatic excision (TMpE) represents a promising surgical strategy for pancreatic head PDAC, offering improved locoregional clearance and the potential for superior oncological outcomes. Our findings, consistent with the current literature, suggest that TMpE-PD provides oncological advantages without increasing the risk of postoperative morbidity. However, the optimal extent of TMpE remains undefined, and further high-quality, multi-center studies with standardized protocols are warranted to establish international consensus and strengthen the evidence base. In conclusion, TMpE demonstrated non-significant trends toward higher R0 resection rates and greater lymph node yields compared with conventional PD. While these findings suggest potential oncologic benefit, definitive conclusions cannot be drawn from this dataset. Further multi-center prospective studies with standardized protocols are necessary.

## Figures and Tables

**Table 1:** Patients’ demographic and clinicopathological characteristics.

	All (*n* = 41)	TMpE-PD (*n* = 17)	Co-PD (*n* = 24)	*p* Value
Age (years)	64.6 ± 10.5	66.4 ± 10.8	63.4 ± 10.3	0.382
Sex				
Female	16 (39.0)	5 (29.4)	11 (45.8)	0.288
Male	25 (61.0)	12 (70.6)	13 (54.2)	
Comorbidity	31 (75.6)	13 (76.5)	18 (75.0)	0.914
Smoking	20 (48.8)	9 (52.9)	11 (45.8)	0.654
BMI (kg/m^2^)	25.0 (23.0–27.0)	25.4 (22.9–27.0)	24.6 (23.0–27.8)	0.952
Preoperative biliary drainage	25 (61.0)	11 (64.7)	14 (58.3)	0.680
Neoadjuvant chemotherapy	1 (2.4)	-	1 (4.2)	1.000
Vascular resection (Venous)	5 (12.2)	2 (11.8)	3 (12.5)	1.000
Operation duration (minutes)	480 (400–495)	485 (480–495)	423 (398–485)	0.067
R status				
R0	21 (51.2)	10 (58.8)	11 (45.8)	0.412
R1	20 (48.8)	7 (41.2)	13 (54.2)	
Pathological stage				
Stage I	3 (7.3)	-	3 (12.5)	0.250
Stage II	18 (43.9)	7 (41.2)	11 (45.8)	
Stage III	20 (48.8)	10 (58.8)	10 (41.7)	
Stage IV	-	-	-	
EBL (ml)	250 (150–300)	250 (150–350)	250 (188–300)	0.139
Number of removed lymph nodes	29.0 (23.0–38.0)	30.0 (25.0–39.0)	26.0 (21.8–36.3)	0.757
Pancreatic duct diameter (mm)	4.0 (3.0–6.0)	4.0 (3.0–5.0)	3.5 (3.0–6.3)	0.788
Pancreatic gland texture				
Firm	5 (12.2)	1 (5.9)	4 (16.7)	0.245
Moderate	32 (78.0)	13 (76.5)	19 (79.2)	
Soft	4 (9.8)	3 (17.6)	1 (4.2)	

**Abbreviations:** TMpE: Total mesopancreatic excision, PD: Pancreaticoduodenectomy, Co: Conventional, BMI: Body Mass Index, EBL: Estimated blood loss, BMI: Body mass index. Data presented as mean ± standard deviation, median (first-third quartiles) or n (%).

**Table 2:** Postoperative outcomes.

All (*n* = 41)	TMpE-PD (*n* = 17)	Co-PD (*n* = 24)	*p* Value
All complications 26 (63.4)	14 (82.4)	12 (50.0)	0.034
Surgical complications 24 (58.5)	12 (70.6)	12 (50.0)	0.187
Surgical side infections 15 (36.6)	8 (47.1)	7 (29.2)	0.241
Intra-abdominal abscess 3 (7.3)	1 (5.9)	2 (8.3)	1.000
Biliary leakage ^x^ 1 (2.4)	1 (5.9)	-	0.415
Chylous leakage 2 (4.9)	2 (11.8)	-	0.166
POPF ^y^ 5 (12.2)	2 (11.8)	3 (12.5)	1.000
Systemic complications 2 (4.9)	2 (11.8)	-	0.166
Atelectasis 2 (4.9)	2 (11.8)	-	0.166
Cardiac complications 1 (2.4)	1 (2.4)	-	0.415
Complication grade ^z^			
I 12 (29.3)	8 (47.1)	4 (16.7)	0.161
II 7 (17.1)	4 (23.5)	3 (12.5)	
III-A 4 (9.8)	1 (5.9)	3 (12.5)	
III-B 1 (2.4)	-	1 (4.2)	
IV-A -	-	-	
IV-B -	-	-	
V 2 (4.9)	1 (5.9)	1 (4.2)	
≥grade-III complications 7 (17.1)	2 (11.8)	5 (20.8)	0.447
Reoperation 1 (2.4)	1 (5.9)	-	0.415
Length of hospital stay (days) 18 (12–21)	18 (12–21)	19.5 (11.8–23.0)	0.730
30-day readmissions 1 (2.4)	-	1 (4.2)	1.000
90-day mortality 4 (9.8)	1 (5.9)	3 (12.5)	0.629

**Abbrevations:** TMpE: Total mesopancreatic excision, PD: Pancreaticoduodenectomy, Co: Conventional, POPF: Postoperative pancreatic fistula. ^x^ Leakage of hepaticojejunal anastomosis ^y^ ISGPS (grade B/C) clinically relevant pancreatic fistulas ^z^ Patients who experienced more than one complication were classified as a higher-grade complication (Clavien–Dindo classification).

**Table 3:** Univariable analysis of factors associated with complications.

	No Complications (*n* = 15)	Complications (*n* = 26)	OR (95% CI, *p* Value)
Age (years)	64.7 ± 8.87	64.6 ± 11.4	1.00 (0.94–1.06, *p* = 0.988)
Sex			
Female	8 (53.3)	8 (30.8)	-
Male	7 (46.7)	18 (69.2)	2.57 (0.69–9.55, *p* = 0.158)
Comorbidity			
No	2 (13.3)	8 (30.8)	-
Yes	13 (86.7)	18 (69.2)	0.35 (0.06–1.91, *p* = 0.223)
Smoking			
No	9 (60.0)	12 (46.2)	-
Yes	6 (40.0)	14 (53.8)	1.75 (0.48–6.35, *p* = 0.395)
BMI (kg/m^2^)	24.0 (22.9–26.6)	25.2 (23.9–27.9)	1.04 (0.88–1.22, *p* = 0.662)
Preoperative biliary drainage			
No	5 (33.3)	11 (42.3)	-
Yes	10 (66.7)	15 (57.7)	0.68 (0.18–2.57, *p* = 0.571)
Vascular resection			-
No	13 (86.7)	23 (88.5)	
Yes	2 (13.3)	3 (11.5)	0.85 (0.13–5.75, *p* = 0.866)
Operation duration (minutes)	480 (423–488)	468 (400–495)	1.00 (0.99–1.01, *p* = 0.582)
EBL (ml)	250 (175–300)	250 (150–338)	1.00 (0.99–1.01, *p* = 0.465)
Pathological stage			
Stage I–II	10 (66.7)	11 (42.3)	-
Stage III	5 (33.3)	15 (57.7)	2.73 (0.72–10.27, *p* = 0.138)
Pancreatic duct diameter (mm)	5.0 (3.5–7.0)	3.5 (3.0–5.0)	0.75 (0.56–1.00, *p* = 0.053)
Pancreatic gland texture			
Firm	3 (20.0)	2 (7.7)	-
Moderate/Soft	12 (80.0)	24 (92.3)	3.00 (0.44–20.44, *p* = 0.262)
Number of removed lymph nodes	26.0 (22.5–36.0)	29.5 (23.0–37.8)	1.00 (0.95–1.05, *p* = 0.959)

**Table 4:** Multivariable analysis of surgical technique and surgical border for complications.

	No Complications (*n* = 15)	Complications (*n* = 26)	Adjusted OR * (95% CI, *p* Value)
Surgical technique			
Co-PD	12 (80.0)	12 (46.2)	-
TMpE-PD	3 (20.0)	14 (53.8)	4.84 (0.90–25.95, *p* = 0.065)
R status			
R0	8 (53.3)	13 (50.0)	-
R1	7 (46.7)	13 (50.0)	1.14 (0.27–4.86, *p* = 0.862)

**Abbreviations:** * Sex, pathological stage, pancreatic duct diameter and pancreatic gland texture were added to the model.

## Data Availability

The data presented in this study are available on reasonable request from the corresponding author. The data is not publicly available due to privacy and ethical restrictions.
